# New and Emerging Treatments for Generalized Pustular Psoriasis: Focus on IL-36 Receptor Inhibitors

**DOI:** 10.3390/pharmaceutics16070908

**Published:** 2024-07-06

**Authors:** João Vilaça, Orhan Yilmaz, Tiago Torres

**Affiliations:** 1 Instituto de Ciências Biomédicas Abel Salazar, University of Porto, 4050-313 Porto, Portugal; vilacapp@gmail.com; 2 College of Medicine, University of Saskatchewan, Saskatoon, SK S7N 5E5, Canada; orhan.yilmaz@usask.ca; 3 Department of Dermatology, Centro Hospitalar Universitário do Porto, 4099-011 Porto, Portugal

**Keywords:** generalized pustular psoriasis, spesolimab, imsidolimab, HB0034, IL-36 receptor inhibitors, treatment

## Abstract

Generalized Pustular Psoriasis (GPP) is a rare and severe subtype of psoriasis that significantly impacts patients’ quality of life. Until recently, no specific treatment modalities were available, and treatment for GPP followed the guidelines for the treatment of plaque psoriasis, consisting of conventional treatments, such as retinoids, methotrexate, and even biologics, which although effective in some cases, may be associated with significant side effects, necessitating more effective and safe options. The pathophysiology of Generalized Pustular Psoriasis is complex and not fully understood, but there is some overlap with the pathogenesis of Plaque Psoriasis. In GPP, the innate immune system seems to play a more significant role, with the interleukin (IL)-36 pathway being fundamentally involved. Spesolimab and imsidolimab, two recently developed therapeutic agents, target the IL-36 inflammatory pathway by binding to the IL-36 receptor (IL-36R). Both biologics have already been evaluated in phase 1 and 2 clinical trials and have shown promising results in terms of safety and efficacy. IL-36 receptor inhibitors demonstrated great efficacy and good safety profile in the management of patients with GPP, demonstrating their potential to emerge as a leading treatment option. This review aims to explore and summarize the current scientific literature on the most recently developed treatments for GPP.

## 1. Introduction

Generalized Pustular Psoriasis (GPP) is a rare and severe auto-inflammatory skin disease characterized by the acute-onset and generalized appearance of superficial erythematous sterile pustules. It has a life-threatening potential due to its multisystemic nature and past history of plaque psoriasis may be a risk factor for developing GPP [1,2]. GPP affects both men and women and it is more common in adults than in children. Its prevalence varies with ethnicity, being more frequent in the Asian population and less common in the Caucasian population [1,3]. The higher prevalence of GPP in Asians likely results from genetic factors. However, data are limited by varying methodologies, diagnostic inaccuracies, and inconsistent definitions. These challenges make it difficult to draw direct comparisons, so reported estimates should be seen as indicative trends rather than precise figures [4].

Clinically, GPP displays a wide range of variability, as it can present as a relapsing condition marked by recurrent flares without pustulation in between, or, alternatively, as a chronic disease featuring persistent pustular eruptions with exacerbations [5]. It is typically characterized by the eruption of sterile pustules through painful, erythematous skin, spreading and recruiting previously unaffected skin. In some cases, it leads to erythroderma. Patients are visibly ill, exhibiting high-grade fever, malaise, and arthralgia [6,7]. Acute infections, menstruation, and rapid withdrawal of systemic corticosteroids have been proven to trigger acute flares [5].

Extracutaneous manifestations, such as cholangitis and acute renal failure, result from its multisystemic nature and can lead to serious complications [6,7]. In fact, this condition imposes a heavy burden of morbidity, and potential mortality, significantly impacting patients’ quality of life [8].

Since it was described for the first time in 1910 by von Zumbusch, finding an adequate treatment for GPP has been a real challenge [1,6]. Its rarity, which makes clinical trials very scarce and difficult to manage, and the necessity of treatment for both flare control and prevention, are the main obstacles [9]. Until very recently, no specific therapeutic agents or guidelines were available for the treatment of GPP, with treatments commonly following the standard guidelines of plaque psoriasis [1]. Therefore, treatment consisted of conventional options such as retinoids and methotrexate. While they might be effective in favorable courses of GPP, they carry significant side effects and are often insufficient in more severe cases [3,6,10].

With the discovery of new biologic therapies, particularly those approved for the treatment of psoriasis, new therapeutic agents started to be considered for the treatment of GPP, providing new and more adequate options [10]. These new agents targeted cytokines involved in the immune pathways of psoriasis (such as TNF, IL-17, or IL-23), which overlap, at certain points with the GPP pathway, and have demonstrated good effectiveness in people with GPP. However, they must be used with caution, as, paradoxically, they have been reported to induce pustular disease in patients being treated for other conditions [9,10].

The discovery of the importance of the IL-36 pathway in the pathogenesis of GPP led to the development of the first specific therapeutic agent for GPP: spesolimab, an IL-36 receptor antagonist. It was recently approved in Japan, the US, and the EU, after demonstrating very positive results. Other similar therapeutic agents are also being evaluated, as this pathway appears to be the main focus for the development of GPP therapies [8,9,11].

This narrative review aims to comprehensively evaluate the development and clinical potential of novel and emerging therapies for GPP. 

## 2. Method

A bibliographic search was performed using the PubMed, Embase, and Medline databases. The search included original and review articles dating from January 2018 to January 2024, written exclusively in English. The following terms were searched in association: “Generalized Pustular Psoriasis”, “Spesolimab”, “Imsidolimab”, “IL36 inhibitors”, and “Treatment”. The articles were selected based on a careful analysis of their abstract and, subsequently, their full text. A total of 38 articles were consulted, all of which are referenced at the end of this review. Additional data regarding the clinical trials were obtained by consulting the website clinicaltrials.gov (accessed on 31 May 2024). 

## 3. Pathophysiology of GPP

Currently, the understanding of the pathophysiological mechanisms of GPP remains incomplete. According to the European Rare and Severe Psoriasis Expert Network (ERASPEN) definition, although GPP shares substantial cytokine pathway overlap with plaque psoriasis, its genetics and pathophysiology diverge significantly [10,11,12]. 

Plaque psoriasis is an autoimmune condition, involving both innate and adaptive immunopathogenic responses. In contrast, GPP is an autoinflammatory condition, resulting primarily from an innate immune inflammation [12]. Some experts even consider it an example of an autoinflammatory keratinization disease [13]. Its histopathology is characterized by the epidermal infiltration of neutrophils and mononuclear cells, leading to the formation of clinically evident, sterile pustules [14]. 

**Figure 1:**
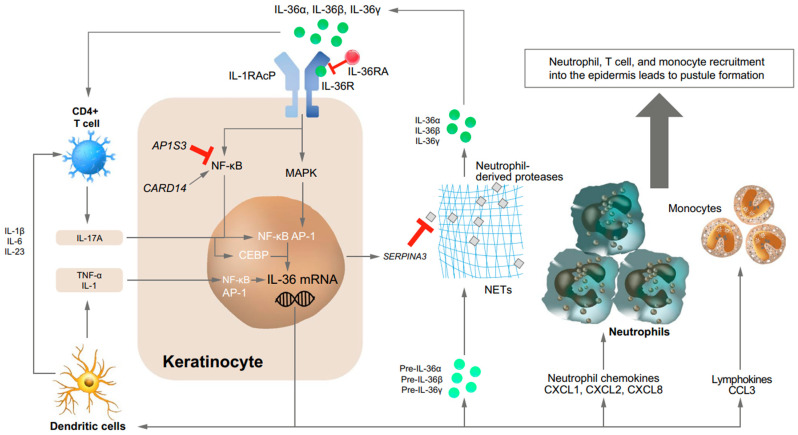
The IL-36 signaling pathway. Keratinocytes secrete IL-36 cytokines as precursors. These are cleaved, becoming biologically active, and bind to IL-36 on the surface of keratinocytes, inducing an inflammatory cascade that promotes IL-36 expression. IL-36 cytokines also induce the expression of numerous cytokines, neutrophilic chemokines, and lymphokines, further propagating this pro-inflammatory cycle. *AP-1*-activating protein-1, *CARD* caspase recruitment domain, *CCL* chemokine (C–C motif) ligand, *CXCL* chemokine (C–X–C motif) ligand, *IL* interleukin, *MAPK* mitogen-activated protein kinase, *mRNA* messenger RNA, *NET* neutrophil extracellular trap, *NFκB* nuclear factor kappa-light-chain-enhancer of activated B cells, *R receptor* RA receptor antagonist, *RAcP* receptor accessory protein, and *TNF* tumor necrosis factor. Image retrieved from Marrakchi et al. 2022 [12]. Licensed under a Creative Commons Attribution-Non-commercial 4.0 International License. License statement: this image is licensed under a Creative Commons Attribution-Non-commercial 4.0 International License, which permits any non-commercial use, sharing, adaptation, distribution, and reproduction in any medium or format, as long as appropriate credit is given to the original author(s) and the source, and a link to the Creative Commons license is provided. To view a copy of this license, visit http://creativecommons.org/licenses/by-nc/4.0/ (accessed on 31 May 2024).

Current scientific evidence supports that the IL-36 axis plays a fundamental role in the innate immune system hyperactivation observed in GPP [15]. IL-36 cytokines are part of the IL-1 superfamily and consist of three pro-inflammatory agonists (IL-36α, IL-36β, IL-36γ) and the IL-36 receptor antagonist. These cytokines are expressed by various cell types, including keratinocytes, epithelial cells, and immune cells, and they act upon these cells in both an autocrine and paracrine fashion [12,15]. They are released as precursors and, to become bioactive, they need to be proteolytically cleaved by specific proteases, particularly neutrophil-derived proteases [15,16]. The three proinflammatory agonists activate nuclear factor-κB (NF-κB) and mitogen-activated protein kinase by binding to the IL-36 receptor (IL-36R). Consequently, downstream pathways are triggered, leading to the production of pro-inflammatory cytokines, chemokines, and co-stimulatory molecules. This process is usually regulated by IL-36Ra, a cytokine that competes with IL-36 agonists for binding to IL-36R, thereby preventing the activation of these pathways (Figure 1) [15].

When IL-36 agonists are excessively expressed or when the function of IL-36Ra is impaired, it triggers a positive feedback loop, leading to uncontrolled signaling and the overproduction of inflammatory cytokines. As a result, chemokines such as CXCL1 and CXCL8, known for their pro-inflammatory properties, are induced. This creates a chemokine gradient that attracts numerous neutrophils to the epidermis, leading to the development of spongiform pustules of Kogoj and the accumulation of neutrophils within the epidermal layer. This manifestation, characterized by the presence of “lakes of pus”, is a hallmark feature frequently observed in patients with GPP [12].

Further data demonstrated that skin biopsy samples of individuals with GPP had higher levels of TNF, IL-1, IL-17, and IL-36 compared to healthy controls. When compared to individuals with plaque psoriasis, the levels of IL-1 and IL-36 were higher, but the levels of IL-17A and interferon-γ were lower [12]. In a significant number of GPP cases, mutations of the IL36RN gene have been identified, indicating a correlation with this condition. This gene is responsible for encoding the anti-inflammatory cytokine IL-36Ra [13,17]. 

Five other genes associated with the development of GPP play roles in the innate immune and autoinflammatory response: CARD14 [18,19], AP1S3 [20], SERPINA3 [21], MPO [22], and TNIP1 [23]. CARD14 mediates NF-κB transduction, contributing to inflammatory signaling [18,19]. AP1S3 loss of function results in autoinflammation by decreasing INF-β expression and elevating IL-1 levels [20]. SERPINA3 inhibits a neutrophil protease, so its loss of function stimulates IL-36 pro-inflammatory activity and neutrophil activation [21]. MPO loss of function leads to MPO deficiency, which fosters the activation of IL-36 signaling by controlling the activity of serine proteases, causing the accumulation and activation of neutrophils [22]. TNIP1 restrains NF-κB signaling, but its gene locus exhibits only a limited association with GPP [16,23].

## 4. Current Management of GPP

At the moment, there are no well-established guidelines for the treatment of GPP due to the lack of evidence, the rarity of the disease, and the spontaneously remitting pattern of its flares [14]. For years, the management of this condition has followed the recommendations established for the treatment of plaque psoriasis [16]. 

### 4.1. Non-Biologic Therapies

The most used therapeutic agents for the management of GPP are still the non-biological ones, particularly retinoids, cyclosporine, and methotrexate. These are considered first-line treatments [24]. 

Retinoids, such as acitretin, are among the oldest systemic agents used to treat GPP. Although many experts consider them a successful treatment modality, the evidence supporting their use is limited. They must be used cautiously due to their teratogenicity predisposition to hepatotoxicity, and other significant side effects [24,25,26]. 

Cyclosporine, a calcineurin inhibitor, is also used in patients with GPP. Although it is effective and useful, the evidence supporting its use is limited to case reports and studies with few subjects. Due to its association with the development of hypertension and renal dysfunction with chronic use, cyclosporine is not recommended as a long-term agent, but is more suitable for controlling acute flares [24,25,26].

Methotrexate, a dihydrofolate reductase inhibitor frequently used in inflammatory conditions, is recommended for the treatment of GPP with some efficacy. However, there are no clinical studies available that prove its effectiveness in managing flares. Additionally, methotrexate has an unfavorable slow onset of action and a safety profile that may not be suitable for some patients, due to its liver toxicity [24,25]. 

There are multiple other non-biologic agents, such as mycophenolate mofetil, hydroxyurea, apremilast, colchicine, and corticosteroids that have been used to some extent in patients with GPP, although the evidence supporting their use is very limited [11,24]. 

Finally, granulocyte and monocyte adsorption apheresis has shown promising results in skin diseases associated with activated neutrophils and is also considered an option for GPP. However, the evidence for its effectiveness is limited to a few case reports [24,27].

### 4.2. Biologic Therapies

In recent years, a better understanding of the pathophysiology of GPP has led to a shift in its treatment towards the greater use of biologics. These biologics have demonstrated high treatment efficacy, and in some cases, have fewer side effects due to their specificity of action [8,24,25]. 

TNF is an important cytokine in the control and amplification of inflammatory pathways and is already used as a molecular target for the treatment of plaque psoriasis [24,25]. Infliximab, etanercept, adalimumab, and certolizumab are known anti-TNF agents that have been shown to be effective and safe in the management of GPP, based on case reports and clinical trials conducted with a small sample of patients. However, in a few cases, paradoxical GPP developed during treatment with adalimumab, infliximab, and certolizumab [26,28,29]. 

IL-17, a cytokine produced by T-helper 17 cells, contributes to the onset of psoriasis by activating keratinocytes, attracting neutrophils, and promoting neovascularization. IL-17A is the most prominent member of this cytokine family [24,30]. Brodalumab, secukinumab, and ixekizumab target the IL-17 pathway: brodalumab by binding to IL-17RA and secukinumab and ixekizumab by inhibiting IL-17A. Small clinical trials conducted with these therapeutic agents have shown good effectiveness in the treatment of GPP, along with a rapid onset of action and an excellent safety profile [30,31,32].

IL-23 plays a crucial role in the development of plaque psoriasis by stimulating Th17 cells, promoting disease progression, and activating keratinocytes [25]. The biologics used to target this cytokine are guselkumab, Risankizumab, and ustekinumab (which also targets IL-12). Data on the use of Guselkumab and risankizumab for GPP are scarce, but a small clinical trial and some case reports have demonstrated their clinical efficacy and safety [24,28,33,34]. Ustekinumab has not been assessed in clinical trials for patients with GPP; its effectiveness has only been reported in a few case reports [8,24,28].

IL-1 is highly involved in the autoinflammation associated with GPP [25]. Canakinumab, gevokizumab, and anakinra are the available therapeutic options for targeting this pathway. Evidence of their use in patients with this condition is limited to a few case reports that have demonstrated encouraging results. However, clinical trials are needed to confirm their efficacy and safety as a viable therapy [24,26]. 

Despite none of these treatments being currently approved in Europe and the United States, Japan has approved the following therapies for GPP after small clinical trials: Bimekizumab [35], Adalimumab, Certolizumab pegol, Infliximab, Secukinumab, Ixekizumab, Brodalumab, Guselkumab, and Risankizumab [24].

## 5. New Treatments for GPP

The discovery of the central role of the IL-36 pathway in the pathophysiology of GPP led to the development of therapeutic agents targeting this immune pathway, such as spesolimab and imsidolimab [11].

### 5.1. Spesolimab

Spesolimab is the first agent developed to target the IL-36 proinflammatory pathway. It consists of a humanized monoclonal immunoglobulin G1 antibody that targets the IL-36R. By binding to this receptor, spesolimab blocks it, preventing the IL-36 ligands from activating it and thereby inhibiting the inflammatory response [36,37]. Currently, spesolimab has been approved by the FDA, EMA, and Japan for the treatment of GPP flares. In addition to GPP, it is also being studied for use in other autoimmune conditions, such as ulcerative colitis, Crohn’s disease, palmoplantar pustulosis, and hidradenitis suppurativa [37].

Spesolimab’s safety and efficacy were first evaluated in a phase I clinical trial involving seven patients with an ongoing GPP flare, who were given a single IV dose of spesolimab (Table 1). Initially, all patients had a Generalized Pustular Psoriasis Physician Global Assessment (GPPGA) score of 3 (moderate disease) By Week 1, five of the patients had already reached a score of 0 or 1 (clear or almost clear skin). By Week 4, all of them had achieved the same results and maintained them until the 20th week. None of the adverse effects were considered serious [38,39].

Effisayil 1, a phase II, multicenter, randomized, double-blind, placebo-controlled clinical trial, involved 53 patients with a GPP flare (Table 1). The patients were randomly assigned in a 2:1 ratio to either the spesolimab group, receiving a single 900 mg IV dose of spesolimab, or the placebo group. After Week 1, patients in both groups had the option to receive a dose of spesolimab on Day 8 as rescue medication, or both [40].

To assess spesolimab’s efficacy in the treatment of GPP, the investigators proposed evaluating the patients’ GPPGA pustulation subscore and GPPGA total score. The primary outcome was a GPPGA pustulation subscore of 0 at Week 1, and the key secondary outcome was a GPPGA total score of 0 or 1 at Week 1. Both scores range from 0 to 4, with higher scores indicating more severe disease [40].

At the beginning of the trial, all the patients had a GPPGA pustulation subscore of at least 2 and a GPPGA total score of at least 3 (moderate-to-severe intensity). By the end of Week 1, 54% of the patients in the spesolimab group had a GPPGA pustulation subscore of 0, and 43% had a GPPGA total score of 0 or 1 This response was significantly higher compared to the placebo group, where only 6% achieved a GPPGA pustulation subscore of 0 and 11% achieved a GPPGA total score of 0 or 1 [40].

On Day 8, 83% of the placebo-randomized patients received a rescue dose of spesolimab, while only 34% of the patients who had already been administered spesolimab needed a second dose This made it impossible to further compare the effects of spesolimab and the placebo. Among the placebo-assigned patients who received a spesolimab rescue dose on Day 8, 73% had reached a GPPGA pustulation subscore of 1 by Week 2, and 53% had reached a GPPGA total score of 0 or 1 [39,40].

Very recently, Effisayil 2, another multicenter, randomized, placebo-controlled, phase 2b trial involving 123 patients with a history of frequent flares and a GPPGA score of 0 or 1 at screening, published its results on evaluating the efficacy and safety of spesolimab for GPP flare prevention (Table 1). These patients were randomly assigned in a 1:1:1:1 ratio to a subcutaneous placebo group, a subcutaneous low-dose spesolimab group (300 mg loading dose followed by 150 mg every 12 weeks), a subcutaneous medium-dose spesolimab group (600 mg loading dose followed by 300 mg every 12 weeks), and a subcutaneous high-dose spesolimab group (600 mg loading dose followed by 300 mg every 4 weeks) The patients were followed for over 48 weeks [41].

To assess spesolimab’s efficacy in preventing GPP flares as a maintenance treatment, this clinical trial analyzed the patients’ time to develop the first flare by week 48, attempting to demonstrate a non-flat dose-response curve. If this was achievable, the secondary objective would be to evaluate the possible superiority of high-dose or medium-dose spesolimab over placebo. This evaluation would also involve analyzing the time to develop the first flare by week 48 and the occurrence of at least one GPP flare by the same time [41].

By Week 48 of the trial, 35 of the original 123 patients had experienced GPP flares: 16 in the placebo group (52%), 7 in the low-dose group (23%), 9 in the medium-dose group (29%), and 3 in the high-dose group (10%). These data demonstrated a non-flat dose–response relationship with statistical significance when comparing spesolimab to the placebo. It was also statistically proven that high-dose spesolimab, compared to the placebo, improved the time to GPP flare and reduced the risk of its occurrence. However, the same improvement was not observed with lower doses of spesolimab [41].

Patients who completed both trials were then eligible to participate in Effisayil ON trial, a long-term extension trial that is assessing the long-term safety and efficacy of spesolimab in patients with GPP. To date, no results from this trial have been published [42]. In Japan and China, phase III trials for spesolimab have already been conducted, but no results have been published yet [43,44].

Regarding safety, GPP patients showed good tolerance to spesolimab. In the Effisayil 1 trial, by week 12, 82% of the patients who received at least one dose of spesolimab had experienced some kind of adverse event (AE), but only 12% had experienced a serious one. Moreover, 66% of patients in the spesolimab group and 56% in the placebo group reported AEs during the first week. Infections were the most frequent AE, occurring in 17% of patients receiving spesolimab compared to 6% in the placebo group. Other common AEs included headache, asthenia and fatigue, and injection-site reactions, most of which were mild-to-moderate in intensity and did not require treatment discontinuation. In the Effisayil 2 trial, the incidence of AEs was similar between patients receiving spesolimab (90%) and those receiving a placebo (87%) over a 48-week period. The incidence of these AEs was also shown not to follow a dose-dependent pattern. The most common AEs reported were pustular psoriasis, which occurred in 25% of patients receiving spesolimab compared to 53% in the placebo group, followed by injection-site erythema (14% vs. 3%). While most AEs were non-serious and non-severe, a greater proportion of patients receiving spesolimab experienced serious AEs (10% vs. 3% in the placebo group), including conditions such as viral encephalitis, pneumonia, and skin bacterial infections [39,40,41].

### 5.2. Imsidolimab (ANB019)

More recently, another therapeutic agent has been designed to treat GPP by targeting the IL-36 pathway. Imsidolimab is a humanized IgG4 monoclonal antibody with high affinity to IL-36R. By binding to this receptor, it inhibits IL-36 activity [11,45,46].

To assess imsidolimab’s clinical efficacy, tolerability, and safety, the GALLOP trial was designed (Table 2). This single-arm, open-label, multiple-dose study enrolled a total of eight patients with relevant GPP. Participants received a single 750 mg IV dose of imsidolimab on the first day of the trial, followed by three subcutaneous doses of 100 mg of the same drug, on Days 29, 57, and 85 [46].

The primary endpoint of the trial was to evaluate the number of patients clinically responding to the treatment at Weeks 4 and 16 after the first administration, using the Clinical Global Impression (CGI) scale. A clinical response was considered positive if the scores were “very much improved”, “much improved”, or “minimally improved” [46].

By Weeks 4 and 16, it was evident that 75% of the patients had achieved clinical response, with 50% even achieving the “very much improved” score. This indicated that imsidolimab acted effectively, rapidly improving the patients’ GPP status [46].

As a secondary outcome, it was also noted that the patients’ quality of life improved [46].

Regarding its safety and tolerability, 75% of the patients reported at least one adverse effect related to imsidolimab, with 2 patients experiencing severe ones. Overall, the doses administered appeared to be adequate and tolerable [46].

The phase 3 clinical trial GEMINI-1 revealed that 53.3% of patients receiving a single 750 mg IV dose of imsidolimab achieved clear or almost clear skin by Week 4, compared to the 13.3% response rate in the placebo group (*p* = 0.0131) (Table 2). Importantly, the trial demonstrated a strong safety profile for imsidolimab, with no severe adverse events, a low incidence of infections comparable to placebo, and no cases of DRESS or Guillain-Barre syndrome. Furthermore, only one patient developed non-neutralizing anti-drug antibodies [47].

### 5.3. HB0034 

Currently, another IL-36R antagonist, HB0034, has been developed and is being tested for use in patients with GPP [48]. 

In a phase Ia study, 44 subjects received HB0034 and 12 received a placebo, with 78.6% reporting treatment-emergent adverse events (TEAEs), nearly all of which were mild, and no serious or severe TEAEs were observed (Table 3). Pharmacokinetics of HB0034 displayed linear properties with an effective half-life of approximately 21 days [49]. 

In a phase Ib study involving three GPP patients, 66.7% achieved a clear/almost clear Generalized Pustular Psoriasis Physician Global Assessment (GPPGA) score at Week 1, and 66.7% had no visible pustules by Week 2 and Week 4 (Table 3). Significant improvements were noted in various clinical measures, including GPPASI, BSA, JDA index score, PSS, DLQI, and CRP. It was reported that the safety was acceptable [49].

### 5.4. Other Therapeutic Agents

The search for new and more treatments for GPP is far from over. Currently, other agents like Zasocitinib (also known as TAK-279), an oral allosteric inhibitor of TYK2 and JNJ-2113 (also known as JNJ-77242113, and previously PN-235), an oral IL-23R antagonist peptide, are in clinical development for GPP [50,51]. 

Information on ongoing clinical trials with these drugs is summarized in Table 4.

**Table 2:** Efficacy and safety data of imsidolimab (ANB019).

Trial	Study Design	Endpoints	Main Results	Main AEs
Phase II (Gallop) [46]	- Single arm, open label - 8 participants - 24 weeks - SD of 750 mg IV at baseline, followed by maintenance 100 mg SC dose monthly	Primary: - Clinical response at Weeks 4 and 16, evaluated using the CGI scale Secondary: - Change in DLQI from baseline	- 75% of the patients achieved clinical response by Weeks 4 and 16, as evaluated by the CGI scale DLQI had reduced 4.1 points by Week 4 and 6.1 points by Week 16	- Blood and lymphatic system disorders - GI disorders - Infections - Respiratory disorders - Skin and subcutaneous disorders - 2 experienced SAEs, resolved without sequelae
Phase III (GEMINI1) [47,52]	- Randomized, PC, DB - 45 participants - 4 weeks - SD of 750 mg IV	Primary: - GPPPGA 0/1 (clear or almost clear) at Week 4	- 53.3% of patients achieved clear or almost clear skin by Week 4, compared to the 13.3% response rate in the placebo group (*p* = 0.0131)	- No severe adverse events, a low incidence of infections comparable to placebo, and no cases of DRESS or Guillain-Barre syndrome. - One patient developed non-neutralizing anti-drug antibodies

AE—Adverse Event; DB—Double Blind; DLQI—Dermatology Quality of Life Index; GI—Gastrointestinal; GPP—Generalized Pustular Psoriasis; GPPGA—Generalized Pustular Psoriasis Physician Global Assessment; CGI—Clinic Global Impression Scale; IV—Intravenous; PC—Placebo Controlled; SAE—Serious Adverse Event; SC—Subcutaneous; SD—Single Dose; DRESS - Drug Reaction with Eosinophilia and Systemic Symptoms.

**Table 3:** Efficacy and safety data of HB0034.

Trial	Study Design	Endpoints	Main Results	Main AEs
Phase Ia [49,53]	- Randomized, PC, DB - 44 participants - SD-Escalation doses from 0.03 mg/kg to 15 mg/kg	- To evaluate the safety, tolerability, and pharmacokinetics in healthy volunteers	- Pharmacokinetics displayed linear properties with an effective half-life of approximately 21 days	- 78.6% of participants reported treatment-emergent adverse events (TEAEs), nearly all of which were mild, and no serious or severe TEAEs observed. - No correlation between the incidence of TEAEs and the dosage administered
Phase Ib [48,49]	- Single arm, open label, Multicenter - 3 participants - 4 weeks	- To evaluate the safety and efficacy in controlling the moderate-to-severe acute GPP flare	- 66.7% achieved a clear/almost clear GPPGA score at Week 1, and 66.7% had no visible pustules by Week 2 and Week 4. - Significant improvements were noted in various clinical measures, including GPPASI, BSA, JDA index score, PSS, DLQI, and CRP	Acceptable safety

AE—Adverse Event; DB—Double Blind; DLQI—Dermatology Quality of Life Index; GPP—Generalized Pustular Psoriasis; GPPGA—Generalized Pustular Psoriasis Physician Global Assessment; GPPASI—Generalized Pustular Psoriasis Area and Severity Index; BSA—Body Surface Area; JDA—Japanese Dermatological Association; PSS—Psoriasis Symptom Scale; PC—Placebo Controlled; TEAE—Treatment-Emergent Adverse Event; SD—Single Dose.

**Table 4:** Ongoing/planned clinical trials for GPP treatments.

Drug Name	Clinical Trial	Phase	Status
Spesolimab	NCT03886246	2	Active, non-recruiting
	NCT05239039	3	Completed
	NCT05200247	3	Completed
	NCT06013969	4	Recruiting
	NCT05670821	Observational cohort	Recruiting
Imsidolimab (ANB019)	NCT05366855	3	Active, non-recruiting
	NCT05352893	3	Completed
HB0034	NCT05064345	1a	Completed
	NCT05512598	1b	Completed
	NCT06231381	2	Recruiting
Zasocitinib (TAK-279)	NCT06323356	3	Recruiting
JNJ-2113 (JNJ-77242113, and previously PN-235)	NCT06295692	3	Recruiting

## 6. Conclusions

Although rare, GPP is a highly incapacitating and potentially life-threatening condition that can affect people worldwide, regardless of age, gender, or ethnicity. For years, its treatment has followed the guidelines for plaque psoriasis, utilizing both biological and non-biological approaches. However, these treatments have not provided optimal results, especially in more severe cases.

Developing new treatments for Generalized Pustular Psoriasis (GPP) is highly challenging due to the disease’s rarity and complexity. GPP is a severe, potentially life-threatening condition marked by extensive pustules and intense systemic inflammation. Its rarity limits the number of patients available for clinical trials, making it difficult to collect sufficient data on the safety and effectiveness of potential treatments. Moreover, the intricate pathophysiology of GPP, involving multiple inflammatory pathways, complicates the development of targeted therapies.

Recently, it was discovered that IL-36 plays a crucial role in the pathogenesis of GPP, triggering the inflammatory cascade that leads to this condition. Consequently, spesolimab and imsidolimab, IL-36 receptor inhibitors, were developed specifically to treat GPP. After undergoing clinical trials, these drugs have been proven to be relevant advancements, having already been approved for the treatment of GPP flares.

In summary, IL-36 receptor inhibitors have shown promising results and are changing the treatment paradigm for GPP. However, further trials are required to confirm their efficacy and safety profile, to assess their role in specific subpopulations, and to evaluate their potential as effective long-term treatments for GPP.

## Figures and Tables

**Table 1:** Efficacy and safety data of spesolimab.

Trial	Study Design	Endpoints	Main Results	Main AEs
Phase I [38,39]	- Single-arm, open-label - 7 participants - Follow-up for 20 weeks - SD of 10 mg/kg IV at baseline	Primary: - Safety and tolerability (% AE) Secondary: - GPPGA 0/1 at week 2	- GPPGA 0/1 in 71% of patients by Week 1 and in 100% of patients by Week 4, sustained up to Week 20	- Mild to moderate eosinophilia - Infections - Vomiting - Pain - Infusion-related reactions - No severe AE
Phase II (Effisayil 1) [39,40]	- Randomized, PC, DB - 53 participants - 12 weeks - Randomized 2:1 into 900 mg IV SD, or placebo	Primary: - GPPGA Pustulation Subscore 0 at Week 1 Secondary: - GPPGA score of 0 or 1	- GPPGA Pustulation subscore of 0 at Week 1 in 54% of spesolimab and 6% of placebo (*p* < 0.001) - GPPGA total 0/1 in 43% of spesolimab and 11% of placebo (*p* < 0.02)	- Infections - Systemic symptoms - Antidrug antibodies
Phase IIb (Effisayil 2) [39,41]	- Randomized, PC, DB - 123 participants - 48 weeks - Randomized 1:1:1:1 into: LD 600 mg, followed by maintenance 300 mg q4w; LD 600 mg, followed by maintenance 300 mg q12w; LD 300 mg, followed by maintenance 150 mg q12w; or placebo	Primary: - Time to first GPP flare Secondary: - Occurrence of at least 1 GPP flare - GPPGA score of 0 or 1 up to Week 48	- Non-flat dose-response relationship for different groups when compared to placebo - Compared to placebo, high-dose spesolimab showed statistically significant superiority on time to GPP (*p* = 0.0005)	- Infections - Injection-site erythema - Arthralgias - Spesolimab and placebo groups with similar incidence of AEs - Spesolimab groups with 10% of serious AEs and 3% in placebo group

AE—Adverse Event; DB—Double Blind; DLQI—Dermatology Quality of Life Index; GPP—Generalized Pustular Psoriasis; GPPGA—Generalized Pustular Psoriasis Physician Global Assessment; IV—Intravenous; LD—Loading Dose; PC—Placebo Controlled; q4w—every 4 weeks; q12w—every 12 weeks; SC—Subcutaneous; SD—Single Dose.

## Data Availability

As this is a narrative review, all the data gathered are available in the studies referenced.
